# A Study on the Rheological and Mechanical Properties of Photo-Curable Ceramic/Polymer Composites with Different Silane Coupling Agents for SLA 3D Printing Technology

**DOI:** 10.3390/nano8020093

**Published:** 2018-02-07

**Authors:** Se Yeon Song, Min Soo Park, Jung Woo Lee, Ji Sun Yun

**Affiliations:** 1 Electronic Convergence Materials Division, Korea Institute of Ceramic Engineering and Technology, 101, Soho-ro, Jinju, Gyeongsangnamdo 52851, Korea; 5005tpdus@kicet.re.kr; 2 Department of Materials Engineering, Pusan National University, 2, Busandaehak-ro 63beon-gil, Geumjeong-gu, Busan 46241, Korea; jungwoolee@pusan.ac.kr; 3 Department of Mechanical System and Design Engineering, Seoul National University of Science and Technology, 232, Gongneung-ro, Nowon-gu, Seoul 01811, Korea; pminsoo@seoultech.ac.kr

**Keywords:** ceramic/polymer composites, rheological properties, 3-D printing, surface treatments

## Abstract

Silane coupling agents (SCAs) with different organofunctional groups were coated on the surfaces of Al_2_O_3_ ceramic particles through hydrolysis and condensation reactions, and the SCA-coated Al_2_O_3_ ceramic particles were dispersed in a commercial photopolymer based on interpenetrating networks (IPNs). The organofunctional groups that have high radical reactivity and are more effective in UV curing systems are usually functional groups based on acryl, such as acryloxy groups, methacrloxy groups, and acrylamide groups, and these silane coupling agents seem to improve interfacial adhesion and dispersion stability. The coating morphology and the coating thickness distribution of SCA-coated Al_2_O_3_ ceramic particles according to the different organofunctional groups were observed by FE-TEM. The initial dispersibility and dispersion stability of the SCA-coated Al_2_O_3_/High-temp composite solutions were investigated by relaxation NMR and Turbiscan. The rheological properties of the composite solutions were investigated by viscoelastic analysis and the mechanical properties of 3D-printed objects were observed with a nanoindenter.

## 1. Introduction

Stereolithography (SLA) is a promising 3D printing technology that achieves more precise manufacturing of 3D geometry ceramic parts than other 3D printing methods [1,2]. For manufacturing ceramic parts by the SLA 3D printing method, appropriate rheological behavior, like shear thinning behavior in suspensions, is desirable [3,4]. Although primarily sub-micron powders are used in alumina processing, alumina powders with a nanometer-scale size are more appropriate for 3D printing applications. 3D-printed objects are created by laying down successive layers with less than 100 µm thicknesses, and the use of sub-micron powders seems to negatively effect the 3D printing process. For high-accuracy 3D printing, fine ceramic particles with a size of a nanometer-scale should be uniformly dispersed. Due to poor interaction between the polymer and ceramic particles, the dispersion stability of ceramics in suspensions is poor, and several problems, such as agglomeration and sedimentation, have been observed during the 3D printing process [5]. Ceramic surface modification by a silane coupling agent is a typical method for improving interfacial adhesion and dispersion stability [6,7,8,9,10,11,12]. The use of a nanometer-scaled alumina powder coated with material with better interfacial adhesion with a photopolymer resin has a positive effect on dispersion stability, because the surface areas of better interfacial adhesion increase, due to nano-scale effect.

**Figure 1:**
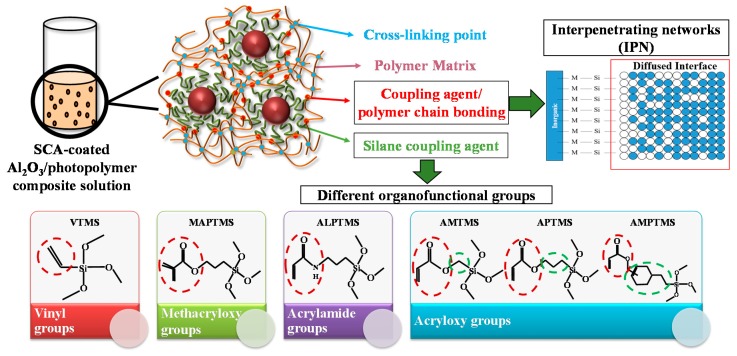
Schematic illustration of dispersion principles of ceramic particles with different organofunctional groups in a photopolymer solution.

The silane coupling agent has two kinds of functional groups, an inorganic functional group and an organic functional group, in the same molecule. Their typical structure is X_3_SiY, where X (methoxy, ethoxy, etc.) is a hydrolysis group and forms a silane-functionalized surface in inorganic materials, and Y (methacryloxy, vinyl, glycidoxy, choloro, etc.) is an organic functional group that enhances the interaction with polymers through the formation of interpenetrating networks (IPNs). Different organofunctional groups in the silane coupling agents create different interfaces and different IPN phenomena between the polymer and ceramic particles, and the ceramic/polymer composites exhibit different rheological behavior and different mechanical properties [13,14,15]. However, in the field of ceramic 3D printing with the SLA method, research on the characteristics of 3D-printed objects by the affinity control of the interface between the polymer and ceramic particles is lacking. In this study, we prepared ceramic particles with various interfacial characteristics by controlling the organofunctional groups of the silane coupling agents, as shown in Figure 1, and then we studied the rheological properties of the ceramic particles in a liquid photopolymer and the mechanical properties of 3D-printed objects.

Al_2_O_3_ ceramic particles were coated with various silane coupling agents (SCAs) with different organofunctional groups by hydrolysis and condensation reactions, and then the SCA-coated Al_2_O_3_ ceramic particles with a coating thickness of about 1.5 nm were dispersed in commercial photopolymers based on the IPN phenomena. Commercial photopolymers (High-temp, Formlabs, Somerville, MA, USA) are mainly composed of acrylated monomers and methacrylated oligomers, and silane coupling agents with different organofunctional groups suitable for the photopolymers and the SLA process were selected. The organofunctional groups based on acryl, such as acryloxy groups, methacrloxy groups, and acrylamide groups, usually have high radical reactivity and are more effective in UV curing systems, and these silane coupling agents seem to promote better adhesion and an improved reinforcing effect. Vinyltrimethoxysilane (VTMS) has the most basic vinyl groups as the organofunctional groups, and acryloxymethyl trimethoxysilane (AMTMS), (acryloxymethyl) phenethyl trimethoxysilane (AMPTMS), and 3-acryloxypropyl trimethoxysilane (APTMS) have the acryloxy groups. Methacryloxypropyl trimethoxysilane (MAPTMS) has the methacryloxy groups, and (acryloxymethyl) phenethyl trimethoxysilane (AMPTMS) has the acrylamide groups. The dispersion stability of the SCA-coated Al_2_O_3_/High-temp composite solutions was investigated by relaxation NMR and Turbiscan. The rheological properties of the ceramic particles in photopolymer solutions were observed by viscoelasticity analysis, and the hardness and elastic modulus of 3D-printed objects with 30 wt % ceramic content were measured by a nanoindenter.

## 2. Experimental Procedures

### 2.1. Materials

All the silane coupling agents, VTMS (C_2_H_6_Si, 233.34 g/mol), AMTMS (C_7_H_14_O_5_Si, 206.27 g/mol), ALPTMS (C_9_H_19_NO_4_Si, 233.34 g/mol), AMPTMS (C_15_H_22_O_5_Si, 310.42 g/mol), APTMS (C_9_H_15_O_5_Si, 234.32 g/mol) and MAPTMS (C_10_H_20_O_5_Si, 248.35 g/mol), were purchased from Gelest (Morrisville, PA, USA). The commercial photopolymer (High-temp) was purchased from Formlabs (Somerville, MA, USA), and commercial γ-aluminum oxide (γ-Al_2_O_3_, 99.5%, 40–50 nm, surface area of 32–40 m^2^/g) was purchased from Alfa Aesar (Haverhill, MA, USA).

### 2.2. Preparation of the SCA-Coated Al_2_O_3_/High-Temp Composite Solutions

For the hydrolysis and condensation reaction, 1 vol % of each silane coupling agent solution was added to the mixed solution of distilled water (91.5 vol %) and ethanol (7.5 vol %), and then the mixture was vigorously stirred for 1 h. A total of 30 wt % of Al_2_O_3_ ceramic particles were added to each silane coupling agent mixture solution, and hydrothermally treated at 100 °C for 3 h. The hydrothermally-treated SCA-coated Al_2_O_3_ ceramic particles were dried at 100 °C for 24 h, and 30 wt % (about 27 vol %) of the SCA-coated Al_2_O_3_ ceramic particles were dispersed in a commercial photopolymer (High-temp) solution based on IPN phenomena with vigorous stirring at room temperature for 72 h.

### 2.3. Characterization of the SCA-Coated Al_2_O_3_ Particles and the SCA-Coated Al_2_O_3_/High-Temp Composite Solutions

The coating morphology and the coating thickness distribution of the SCA-coated Al_2_O_3_ ceramic particles were observed by field-emission transmission electron microscopy (FE-TEM, JEM-2100F, JEOL, Tokyo, Japan). The particle size distribution of the SCA-coated Al_2_O_3_ ceramic particles was measured by a laser scattering particle size distribution analyzer (partica LA-950V2, HORIBA, Kyoto, Kyoto Prefecture, Japan). The initial dispersibility and dispersion stability of the SCA-coated Al_2_O_3_/High-temp composite solutions were investigated by a relaxation NMR (Acorn Area, Xigo Nanotools, Bethlehem, PA, USA) at room temperature and Turbiscan (AGS, Formulacation, Toulouse, France) at room temperature for 72 h. The viscoelasticity of the SCA-coated Al_2_O_3_/High-temp composite solutions was measured by diffusing wave spectroscopy (DWS, Rheolaser master, Formulacation, Toulouse, France) at room temperature, and the viscosity was studied by a rheometer (MCR-502, Anton Paar, Graz, Austria) under the shear rate ranges of 0.1–1000 s^−1^ at room temperature for 48 h. 3D-printing objects with a layer thickness of 100 µm were 3D-printed by a UV laser with an intensity of 0.2 W. The hardness and elastic modulus of the 3D-printed objects were investigated by nanoindentation (iMicro nanoindenter, Nanomechanics, Inc., Oak Ridge, TN, USA) under a target load of 1000 mN and a target depth of 10,000 nm. The hardness of the 3D-printed objects was also observed by a microhardness tester (HM-113, Mitutoyo, Kanagawa, Japan) under a 0.1 kg condition.

## 3. Results and Discussion

### 3.1. Characterization of the SCA-Coated Al_2_O_3_ Ceramic Particles

**Figure 2:**
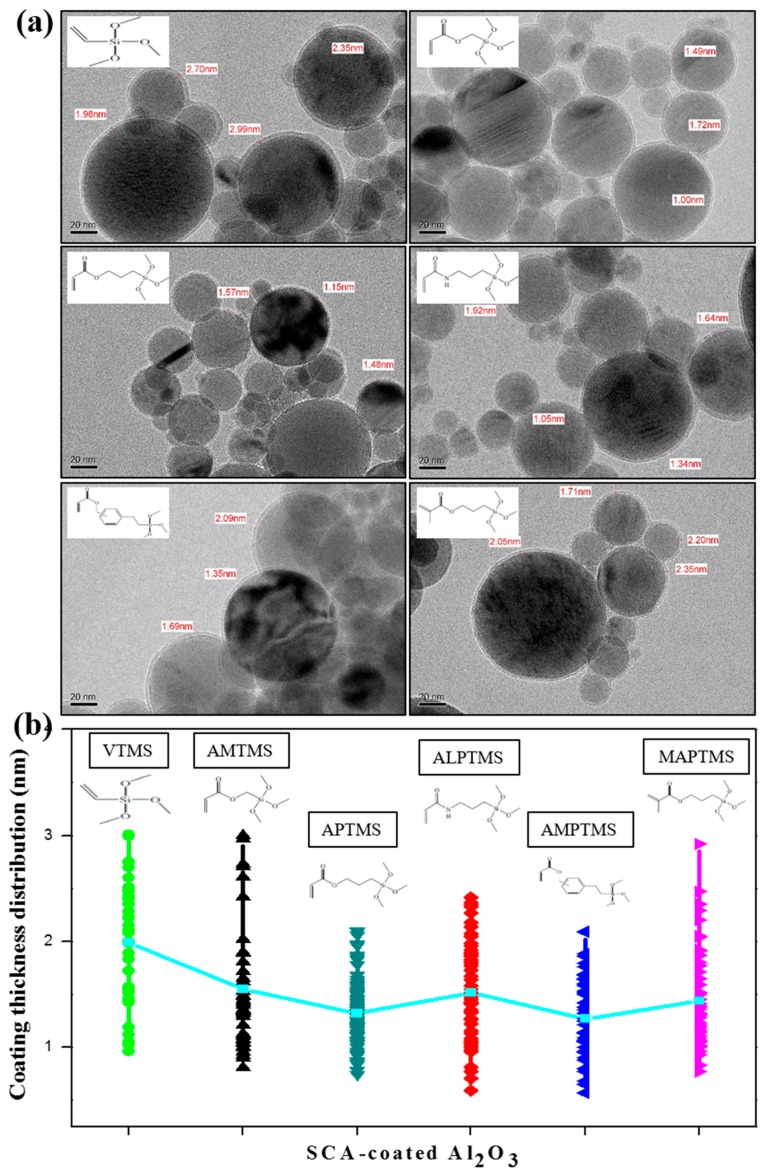
(**a**) TEM images and (**b**) the coating thickness distribution of the SCA-coated Al_2_O_3_ ceramic particles with different organofuctional groups.

The TEM images in Figure 2a revealed that the SCA-coated Al_2_O_3_ ceramic particles were uniformly coated by various SCAs, and Figure 2b shows the coating thickness distribution and average coating thickness of the SCA-coated Al_2_O_3_ ceramic particles calculated based on the TEM images. The average coating thickness of the SCA-coated Al_2_O_3_ ceramic particles was about 1.5 nm, and the coating thickness distribution was between 1 nm and 3 nm. Since the SCA coating reaction on the ceramic surface was mainly determined by the hydrolysis and condensation reaction of the methoxy groups, the coating thickness distributions of various SCAs with the same inorganic functional groups of methoxy groups were similar even though they had different organofunctional groups.

**Figure 3:**
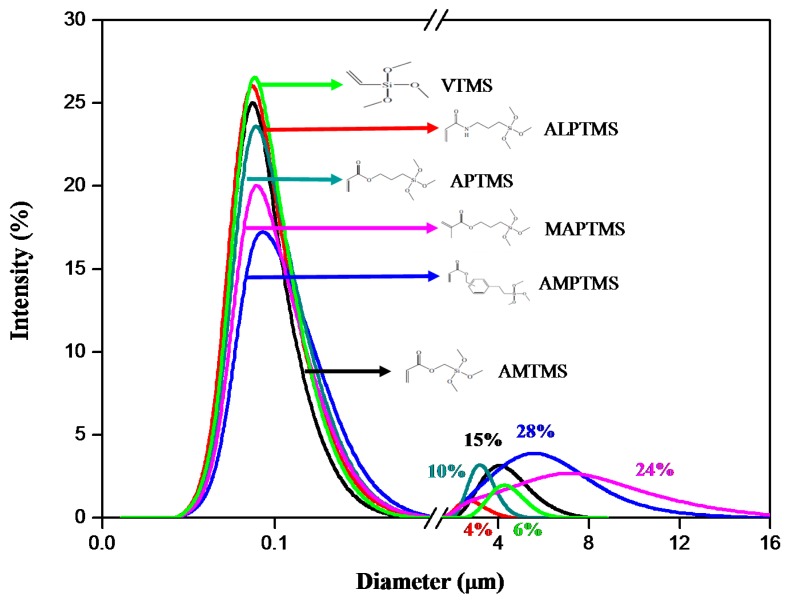
Particle size distribution curves of the SCA-coated Al_2_O_3_ ceramic particles with different organofunctional groups.

The particle size distribution curves of the Al_2_O_3_ ceramic particles coated with SCAs with different organofunctional groups are shown in Figure 3. All kinds of the SCA-coated Al_2_O_3_ particles showed a similar particle size distribution, and all the coated particles had a mode size (diameter of maximum frequency) of about 0.08 µm. In the case of the AMPTMS-coated Al_2_O_3_ particles and MAPTMS-coated Al_2_O_3_ particles, the percentages of the large particles with a diameter range of 1.5–16 µm was 28% and 24%, respectively, and the ratio of large particles was higher than other SCA-coated Al_2_O_3_ particles. In other words, the AMPTMS-coated Al_2_O_3_ particles and the MAPTMS-coated Al_2_O_3_ particles had a larger mean (average) size of about 1.5 μm than other SCA-coated Al_2_O_3_ particles because they tended to agglomerate. The mean size of the VTMS-coated Al_2_O_3_ particles, AMTMS-coated Al_2_O_3_ particles, APTMS-coated Al_2_O_3_ particles and ALPTMS-coated Al_2_O_3_ particles were 0.34 μm, 0.64 μm, 0.40 μm and 0.19 μm, respectively, and the agglomeration tendency of these particles was similar.

### 3.2. Initial Dispersibility and Dispersion Stability of the SCA-Coated Al_2_O_3_/High-Temp Composite Solutions

**Figure 4:**
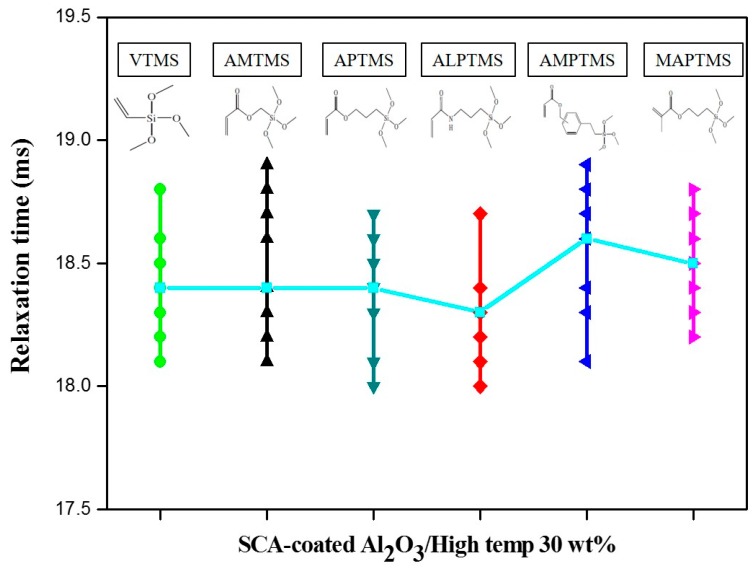
Relaxation times of the SCA-coated Al_2_O_3_/High-temp composite solutions with different organofunctional groups.

To prepare the SCA-coated Al_2_O_3_/High-temp composite solutions, 30 wt % content of SCA-coated Al_2_O_3_ ceramic particles with different SCAs were mixed with a commercial photopolymer solution of High-temp based on IPN phenomena. The initial dispersibility of the SCA-coated Al_2_O_3_/High-temp solutions was investigated by relaxation NMR, as shown in Figure 4. The relaxation NMR shows that the relaxation time was relatively longer in the case of the AMPTMS-coated Al_2_O_3_ particles and MAPTMS-coated Al_2_O_3_ particles because of the strong agglomeration tendency in the particle size distribution curves in Figure 3. The other SCA-coated Al_2_O_3_ ceramic particles, namely, VTMS-coated Al_2_O_3_ particles, AMTMS-coated Al_2_O_3_ particles, APTMS-coated Al_2_O_3_ particles, and ALPTMS-coated Al_2_O_3_ particles, with a similar tendency of the agglomeration in Figure 3 have similar relaxation times, and these results mean that the coated particles have similar rotation and translation motions in the photopolymer solution. In other words, the initial dispersibility of the SCA-coated Al_2_O_3_ ceramic particles in the High-temp solution, except for the AMPTMS-coated Al_2_O_3_ particles and MAPTMS-coated Al_2_O_3_ particles, was similar.

**Figure 5:**
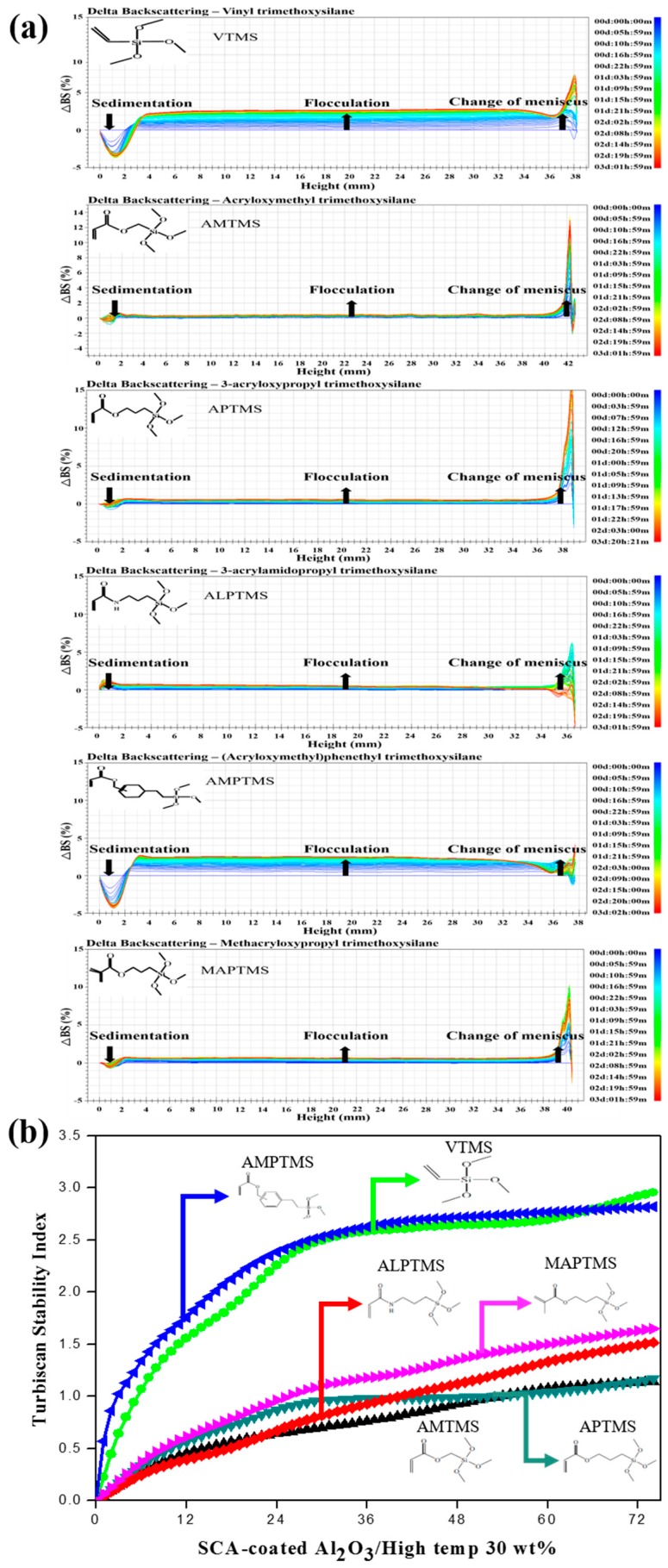
(**a**) Backscatter curves and (**b**) Turbiscan stability index profiles of the SCA-coated Al_2_O_3_/High-temp composite solutions with different organofunctional groups.

The dispersion stability of the SCA-coated Al_2_O_3_/High-temp composite solutions with 30 wt % content of the SCA-coated Al_2_O_3_ ceramic particles with different organofunctional groups was observed by Tubiscan, as shown in Figure 5. For investigation of the dispersion stability by Turbiscan, each sample was placed in a cylindrical glass cell. A detection head composed of a near infrared light source, transmitted light and backscattering detector moves the entire height of the sample up and down, and scans in about 40 μm units to obtain transmittance and backscattering data [16]. The transmittance and backscattering of the pulsed and near infrared light (λ = 880 nm) were measured, but the backscattering profile was studied because the transmitted light of the opaque SCA-coated Al_2_O_3_/High-temp composite solutions was zero. As shown in Figure 5a, the change in backscattering curves (ΔBS) of the SCA-coated Al_2_O_3_/High-temp composite solutions with different organofunctional groups was observed for 72 h. The backscattering profiles indicate that the particle migration (sedimentation, creaming) and particle size (flocculation, coalescence) changed according to the entire sample height. In the all SCA-coated Al_2_O_3_/High-temp composite solutions, changes in sedimentation, flocculation and meniscus were observed in the bottom area, the middle area and the top area, respectively. The VTMS-coated Al_2_O_3_/High-temp composite solution, which had a thin coating thickness, less particle agglomeration and good initial dispersibility, had a thick sedimentation layer and a thick flocculation layer in the backscattering curves. This was because VTMS with the most basic organofunctional groups of vinyl groups had a weak network with the acrylate resin. In the AMTMS with the acryloxy groups, APTMS with the acryloxy groups and ALPTMS with the acrylamide groups, better characteristics were observed like a thin coating thickness, less particle agglomeration, good initial dispersibility and good dispersion stability, and the organofunctional groups of the acryloxy groups and the acrylamide groups contributed to a stable network with the acrylate resin. On the other hand, in the AMPTMS with the acryloxy groups, the backscatter curves of the AMPTMS-coated Al_2_O_3_/High-temp composite solution showed a thick sedimentation layer and a thick flocculation layer due to the strong particle agglomeration in the solution by the benzene located in the middle of the AMPTMS molecule. In the MAPTMS with the methacryloxy groups, although there was strong particle agglomeration and poor initial dispersibility, the dispersion stability in the acrylate resin was good because of the methacryloxy groups making a stable network with the acrylate resin. The dispersion stability by the Turbiscan can be evaluated by Turbiscan Stability Index (TSI) parameters, which is the sum of all the scan differences from the bottom to the top of the measuring cell [17]. As shown in Figure 5b, the VTMS-coated Al_2_O_3_/High-temp composite solution and the AMPTMS-coated Al_2_O_3_/High-temp composite solution, which had thicker sedimentation and thicker flocculation layers, had the highest TSI values, but the other SCA-coated Al_2_O_3_/High-temp composite solutions had similar TSI values.

### 3.3. Rheological Behavior of the SCA-Coated Al_2_O_3_/High-Temp Composite Solutions

**Figure 6:**
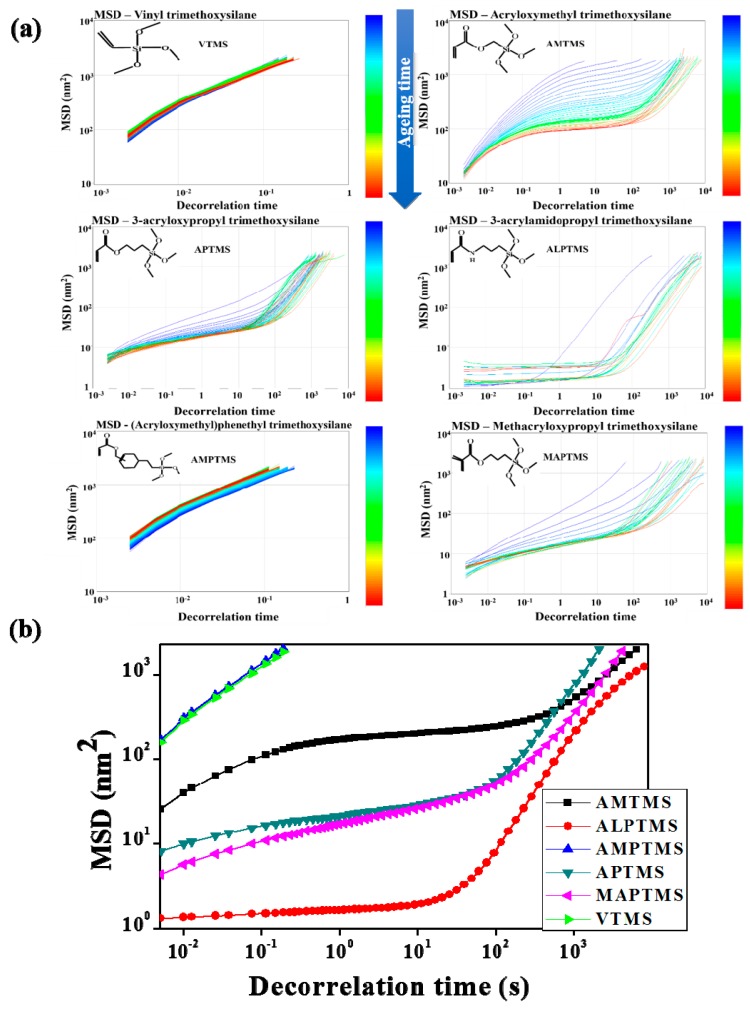
MSD-decorrelation time curves of the SCA-coated Al_2_O_3_/High-temp composite solutions with different organofunctional groups: (**a**) MSD-decorrelation time curves during 48 h and (**b**) MSD-decorrelation time curves at 12 h.

The viscoelastic results measured by diffusing wave spectroscopy (DWS) in Figure 6 show the rheological behavior of the SCA-coated Al_2_O_3_/High-temp composite solutions with 30 wt % content of the SCA-coated Al_2_O_3_ ceramic particles with different organofunctional groups. The DWS method measures the movement of the solution and tracks the interaction of the solvent and particles without damage or modification to the solution. When a laser beam irradiates fluid samples, the photons irradiated in the solution are backscattered by objects like particles suspended in the solution, and a dynamic interference pattern called a speckle image is recorded [18]. Based on the dynamic interference pattern, the mean square displacement (MSD), which denotes the average trajectory by numerous particles, was investigated as a function of time. The MSD-decorrelation time curves of the SCA-coated Al_2_O_3_/High-temp composite solutions with different functional groups during 48 h are shown in Figure 6a, and the MSD-decorrelation time curves at 12 h for comparisons of viscoelasticity behaviors of the various SCA-coated Al_2_O_3_/High-temp composite solutions are shown in Figure 6b. The VTMS-coated Al_2_O_3_/High-temp composite solution and the AMPTMS-coated Al_2_O_3_/High-temp composite solution demonstrated linear graphs and higher MSD values, which means that the solution had purely viscous behavior. In other words, the VTMS-coated Al_2_O_3_ particles and the AMPTMS-coated Al_2_O_3_ particles seem to make a poor network with the High-temp resin and move freely in the resin without interference of polymer chains [19]. The MSD curves of the AMTMS-coated Al_2_O_3_/High-temp composite solution were not linear and there were plateaus at longer times, which mean an increase in viscoelasticity. These results indicate that the acryloxy groups of the AMPTMS-coated Al_2_O_3_ particles make a strong network with the High-temp resin, and the network formation severely restricted the motion of polymer chains [19]. In the APTMS-coated Al_2_O_3_/High-temp composite solution with a longer C-C chain length compared to AMTMS, the change of the MSD curves according to aging time was smaller than that with the AMTMS-coated Al_2_O_3_/High-temp composite solution, and the MSD values of the elastic plateau were lower. In other words, the recovery rate after the initial pre-shear was faster than the AMTMS, and the speckle pattern deformation of MSD was lower. This was because as the chain length of the organofunctional groups became longer, the viscoelastic behavior became stronger, and the network tightened. In the MAPTMS-coated Al_2_O_3_/High-temp composite solution with the methacryloxy groups, although a strong aggregation tendency and relatively poor initial dispersibility were observed, characteristics of dispersion stability and viscoelasticity were almost the same as the APTMS-coated Al_2_O_3_/High-temp composite solution. In the solution, the affinity of the organic functional groups of the particle surface to the polymer seemed to be a more important factor in making stable IPN than the affinity of the particles themselves. In the ALPTMS-coated Al_2_O_3_/High-temp composite solution with the acrylamide groups, the change in the MSD curves according to aging time was the smallest, and MSD curves of the elastic plateau were the longest, and had the lowest values. These results confirm that the movement of particles was not free because the particles were strongly trapped in the network structure.

**Figure 7:**
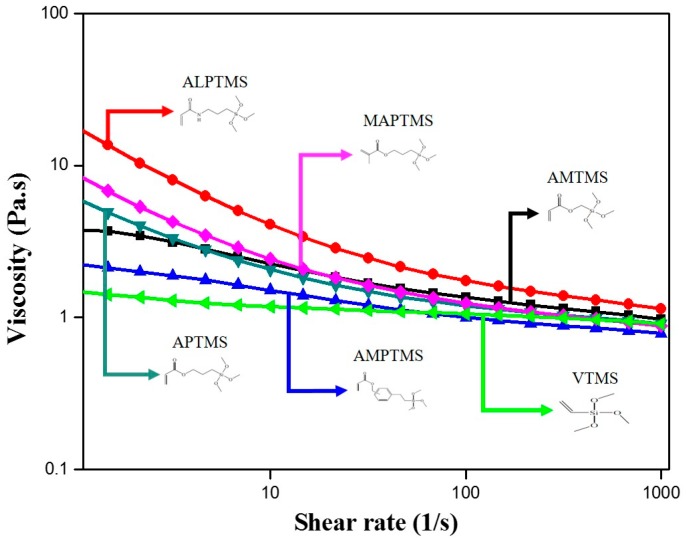
Viscosity curves of the SCA-coated Al_2_O_3_/High-temp composite solutions with different organofunctional groups.

The viscosities of the SCA-coated Al_2_O_3_/High-temp composite solutions with 30 wt % content of SCA-coated Al_2_O_3_ ceramic particles with different organofunctional groups as a function of shear rates are shown in Figure 7. The viscosity of the various SCA-coated Al_2_O_3_/High-temp composite solutions showed greater differences at lower shear rates, but as the shear rate increased, the viscosity of the composite solutions became similar. The VTMS-coated Al_2_O_3_/High-temp composite solution exhibited near-fluid behavior, which was close to Newtonian flow, but shear thinning behavior [20,21,22] was observed in the other SCA-coated Al_2_O_3_/High-temp composite solutions. In the viscoelastic results of Figure 6, the viscosity was higher in the composite solution with stronger viscoelastic behavior, and shear thinning behavior was more clearly observed. In particular, the ALPTMS-coated Al_2_O_3_/High-temp composite solution with the acrylamide groups, which had the strongest viscoelasticity in Figure 6, had the highest viscosity. In this regard, the stronger viscoelastic behavior caused by a strong network with polymer chains seemed to restrict the movement of the polymer chains and increase the viscosity.

### 3.4. Mechanical Properties of the 3D-Printed Objects Printed Using the SCA-Coated Al_2_O_3_/High-Temp Composite Solutions

**Figure 8:**
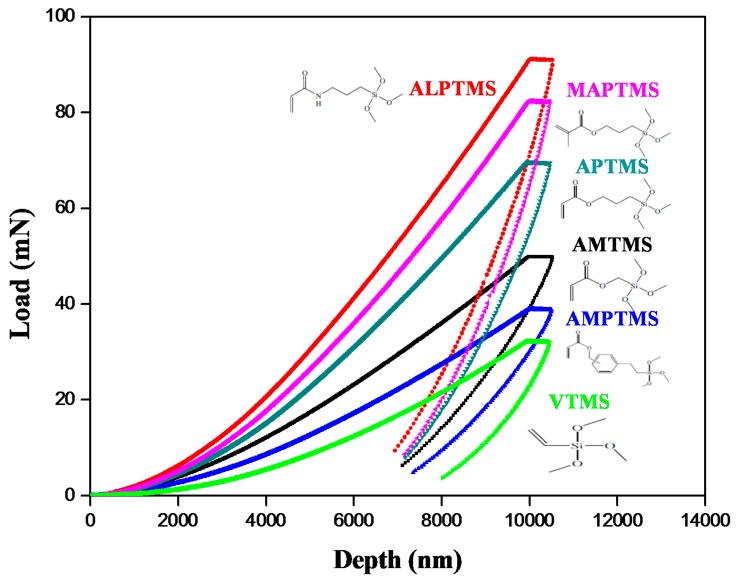
Load-depth curves of the 3D-printed objects printed using the SCA-coated Al_2_O_3_/High-temp composite solutions with different organofunctional groups measured by nanoindentation.

To evaluate the mechanical properties (hardness and elastic modulus) according to the viscoelastic behaviors, the 30 wt % SCA-coated Al_2_O_3_ ceramic particles were dispersed in High-temp resin and printed by a SLA 3D printer. The specimens with a rectangular shape (10 mm × 10 mm × 1 mm) were 3D-printed and analyzed by a nanoindentation and microhardness tester, as shown in Figure 8 and Table 1. The load-depth curves of the 3D-printed objects printed using the various SCA-coated Al_2_O_3_/High-temp composite solutions were measured by nanoindentation, as shown in Figure 8, and the hardness and elastic modulus in Table 1 were calculated based on the load-depth graphs measured by nanoindentation [23]. The 3D-printed objects based on VTMS and AMPTMS, which showed viscous behavior, had the lowest hardness and elastic modulus. The hardness and elastic modulus of 3D-printed objects based on SCA with stronger viscoelastic behavior had the higher values, and 3D-printed objects based on ALPTMS with the strongest viscoelasticity had the highest hardness and the highest elastic modulus. In other words, the stronger the network, the stronger the viscoelastic of the composite solutions. These stronger viscoelastic behaviors seemed to improve the dispersion stability and mechanical properties.

## 4. Conclusions

To improve interfacial adhesion and dispersion stability, various SCA-coated Al_2_O_3_ ceramic particles with different organofunctional groups were prepared through hydrolysis and condensation reactions, and the particles were dispersed in a commercial photopolymer (High-temp) based on IPN phenomena. The coating thickness and morphology of the various SCA-coated Al_2_O_3_ ceramic particles with different organofunctional groups in FE-TEM analysis were similar because they had the same inorganic functional groups of methoxy groups. Turbiscan, relaxation NMR, and viscoelastic analysis results confirmed that the ALPTMS-coated Al_2_O_3_/High-temp composite solution with the acryloxy groups had the best dispersion stability and the strongest viscoelastic behaviors because the particles were strongly trapped in the network structure, and this stronger viscoelasticity improved the mechanical properties of 3D-printed objects.

## Figures and Tables

**Table 1:** Hardness and elastic modulus values of the 3D-printed objects printed using the SCA-coated Al_2_O_3_/High-temp composite solutions with different organofunctional groups.

Sample ID	Nano Indentation Hardness (Gpa)	Nano Indentation Elastic Modulus (Gpa)	Microhardness (Gpa)
**VTMS**	0.014	0.7	0.020
**AMTMS**	0.045	1.3	0.035
**APTMS**	0.053	1.6	0.038
**AMPTMS**	0.030	1.0	0.029
**ALPTMS**	0.074	2.7	0.047
**MAPTMS**	0.061	1.9	0.041
